# Exciton–Phonon Coupling in Single ZnCdSe-Dot/CdS-Rod Nanocrystals with Engineered Band Gaps from Type-II to Type-I

**DOI:** 10.1021/acsphotonics.4c00931

**Published:** 2024-09-06

**Authors:** Florian Johst, Jannik Rebmann, Hans Werners, Lars Klemeyer, Jagadesh Kopula Kesavan, Dorota Koziej, Christian Strelow, Gabriel Bester, Alf Mews, Tobias Kipp

**Affiliations:** † Institute of Physical Chemistry, University of Hamburg, Grindelallee 117, Hamburg D-20416, Germany; ‡ Institute of Nanostructure and Solid State Physics, University of Hamburg, Luruper Chaussee 149, Hamburg D-22761, Germany; § The Hamburg Centre for Ultrafast Imaging, Luruper Chaussee 149, Hamburg 22761, Germany

**Keywords:** nanocrystals, colloidal, exciton−phonon coupling, single-particle spectroscopy, low temperature, band gap engineering

## Abstract

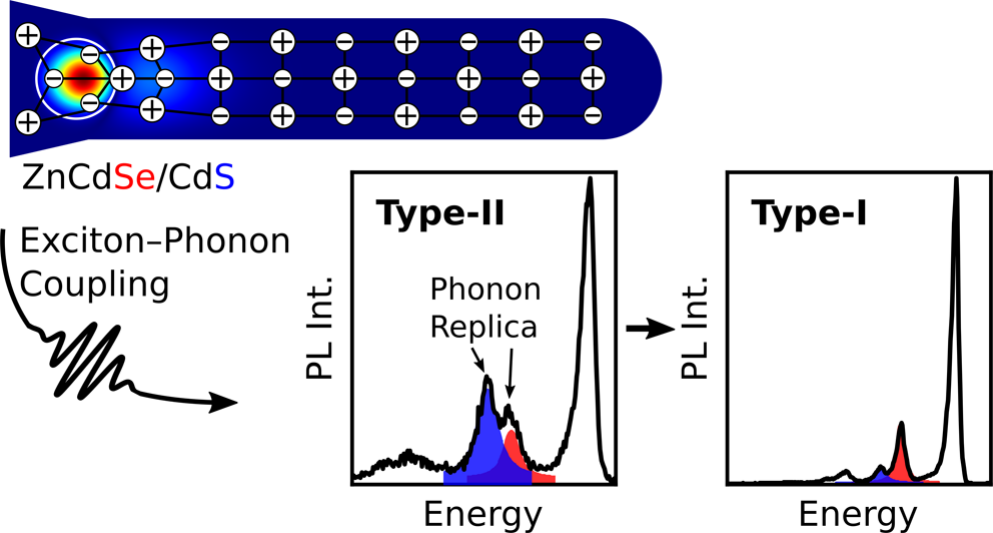

Exciton–phonon coupling limits the homogeneous emission line width of nanocrystals. Hence, a full understanding of this is crucial. In this work, we statistically investigate exciton–phonon coupling by performing single-particle spectroscopy on colloidal Zn_1–*x*_Cd_*x*_Se/CdS and CdSe/CdS dot-in-rod nanocrystals at cryogenic temperatures (*T* ≈ 10 K). In situ cation exchange enables us to analyze different band alignments and, thereby, different charge-carrier distributions. We find that the relative intensities of the longitudinal optical S- and Se-type phonon replicas correlate with the charge-carrier distribution. Our experimental findings are complemented with quantum mechanical calculations within the effective mass approximation that hint at the relevance of surface charges.

## Introduction

Colloidal semiconductor nanocrystals (NCs) are promising building blocks for optoelectronic devices.^1−8^ Their properties can be fine-tuned by controlling their morphologies and composition. Dot-in-rod nanocrystals (DRs) are particularly interesting, as they can be synthesized with narrow size distributions,^9^ high quantum yields,^10,11^ different material combinations, and thus different band alignments.^12−16^ They provide strong polarized emission,^17−20^ have high extinction coefficients,^17^ and can be aligned over several micrometers.^9^ The degree of polarization can be even increased when using elongated cores.^21^ DRs can be used as gain media in lasers,^22−25^ in light-emitting diodes,^26,27^ in catalysis,^28−32^ in biosensing,^33−36^ and optical switches.^37^ The charge-carrier localization in NCs determines their fluorescence energy, fluorescence lifetime, polarization, sensitivity to intended external electric fields, and often unintended influences of ligands and surface charges, as well as the possibility of charge separation for photocatalytic reactions. Charge-carrier localization also influences the coupling to lattice vibrations, which in turn again impacts the optical properties: The coupling is a major contribution to the homogeneous fluorescence line width.^38^ Finding a clear correlation between exciton localization and phonon coupling is essential for understanding the fundamentals of exciton–phonon coupling. Once it has been fully understood, it allows for optimization of NC devices on the one hand and makes it a tool for determining the charge-carrier localization on the other.

Different methods have been used to investigate exciton–phonon coupling in NCs, including Raman spectroscopy,^39,40^ femtosecond pump probe spectroscopy,^41^ fluorescence-line narrowing^42^ and photoluminescence excitation spectroscopy.^43^ However, the experimental methods and theoretical models show discrepancies in the magnitude of exciton–phonon coupling.^44,45^ Therefore, the fundamental understanding of exciton–phonon coupling must be extended.

In this work, we perform single-particle photoluminescence (PL) spectroscopy at low temperatures, which is a perfect method for detailed investigations of exciton–phonon coupling in NCs. It expresses itself in the form of phonon replicas within the spectra.^46^ In contrast to, for example, Raman spectroscopy, PL inherently only probes the region of the particles where the excitonic recombination takes place. The low-temperature measurements allow spectral resolution of the phonon frequencies and intensities, from which the involved material composition can be deduced. The benefit of single-particle spectroscopy is that it circumvents the inhomogeneous broadening of nanocrystal ensembles. By analyzing single-particle spectra measured over time, spurious effects of spectral diffusion and jittering can be eliminated. We developed a unique heteronanostructure system with a gradual change from type-II to type-I band alignment in which the geometry is kept nearly constant. This system is based on alloyed Zn_1–*x*_Cd_*x*_Se/CdS DRs, for which the core and shell phonon energies are well separated. By controlling the Cd fraction *x*, the band alignment and the corresponding exciton localization was manipulated, making the system a suitable test bed for a gradual change of phonon coupling. With this system, we also demonstrate that in situ cation exchange is suitable for band gap engineering in DRs. The exciton–phonon coupling is investigated from over 300 single-particle PL spectra at cryogenic temperatures. We find that the relative intensities of the S- and Se-type LO-phonon replicas reflect the different exciton distributions. The experimental data are compared to theoretical modeling of exciton–phonon coupling. To this end, we performed quantum mechanical calculations of the excitons within the effective mass approximation, including the Coulomb interaction between the electron and hole. Our results show that coupling to different phonon modes depends on the band alignment and that surface charges become more relevant for type-II band alignments. The fundamental knowledge gained from our work on single particles at cryogenic conditions is important for the design of NC devices at room temperature.^38,47^

## Results and Discussion

Four Zn_1–*x*_Cd_*x*_Se/CdS DR samples (labeled DR-1 to DR-4) with different *x* were synthesized (for details see Supporting Information). Starting point for samples DR-1 to DR-3 is the same batch of presynthesized ZnSe NCs exhibiting an average diameter of *d* = 3.16 nm. A dispersion of these ZnSe NCs as well as a S-precursor solution were separately hot-injected into a Cd-precursor solution at 320 °C. For sample DR-1, ZnSe NCs and the S-precursor were injected simultaneously (Δ*t* = 0 s), while for sample DR-2 and DR-3, the S-precursor injection was delayed with respect to the ZnSe NCs injection by Δ*t* = 10 s and Δ*t* = 60 s, respectively. The increasing injection delay should increase the degree of cation exchange occurring in the original ZnSe NCs before CdS-shell growth. After complete shell formation, further cation exchange inside the core is inhibited. Hence, we expect samples DR-1 to DR-3 to have increasing Cd fractions *x* in the core. As a reference, sample DR-4 represents pure CdSe/CdS DRs (i.e., *x* = 1) that have been synthesized from CdSe NCs (diameter *d* = 3.18 nm).

**Figure 1:**
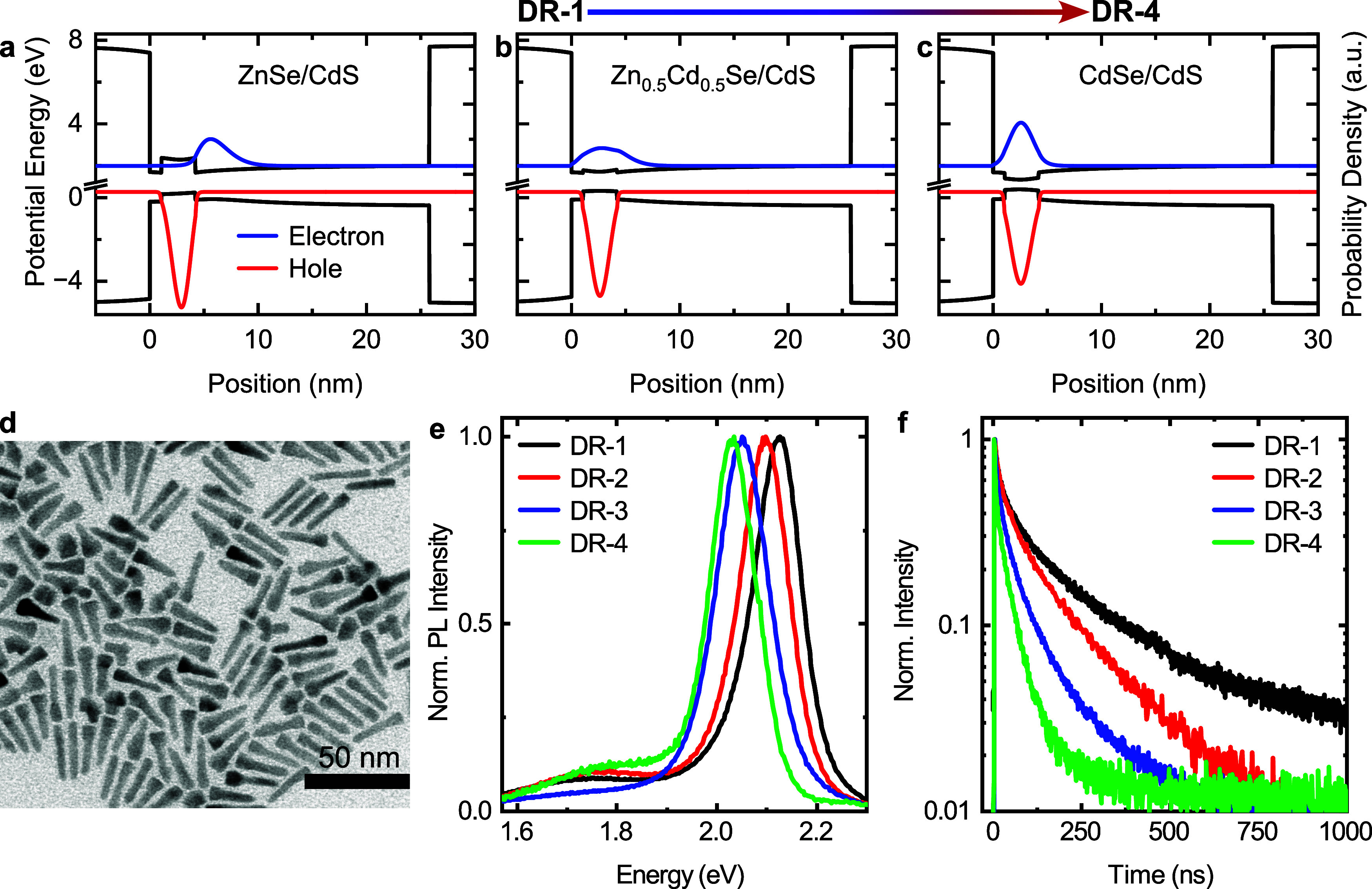
(a–c) Cross sections of the calculated potential energies (black) and probability densities of electron (blue) and hole (red) along the DR axis. (d) TEM image of Zn_1–*x*_Cd_*x*_Se/CdS DRs synthesized without precursor-injection delay of the S-precursor. (e) Ensemble photoluminescence spectra and (f) time-resolved PL decays measured at *T* ≈ 10 K.

Figure 1a–c illustrates results of model calculations within the effective mass approximation, showing the theoretical range of band alignments and exciton localizations (see SI). A rising Cd fraction results in a lowered conduction band offset and, thus, in a larger electron–hole wave function overlap and a reduced exciton energy. Figure 1d shows a representative transmission electron microscopy (TEM) image of sample DR-1 (see Figures S1–S3 for TEM images of other samples). The visibly thickened side is known to form around the cores in ZnSe/CdS DRs.^12,48^ This feature is also present in the CdSe/CdS DRs sample (DR-4), possibly resulting from the use of cores with an initial zinc blende phase. Table 1 gives the average diameters of the DRs at the center (*D*) and at the thickened side (*C*) as well as their average length (*L*). Samples DR-1 to DR-4 exhibit similar geometries: *D* and *L* overlap in their standard deviations. Figure 1e depicts normalized ensemble PL spectra of samples DR-1 to DR-4 at *T* ≈ 10 K. The main PL peak red-shifts by about 100 meV in energy from DR-1 to DR-4 (Table 1). All samples show a broad low-energy band around 1.75 eV with low intensity, indicating trap emission.^49,50^ The time-resolved PL data in Figure 1f reveal decreasing fluorescence lifetimes for the sample series. Fitting the decay curves with biexponential functions yields average PL lifetimes between 172 and 34 ns (Table 1). Both the decrease in PL-emission energy and PL lifetime can be explained by an increasing Cd fraction *x* within the cores, thus with a transition from type-II to type-I band alignment.

**Table 1:** Characteristics of the Sample Series^a^

sample	core	Δ*t* (s)	*D* (nm)	*C* (nm)	*L* (nm)	*E*_PL_ (eV)	*τ*_ave_ (ns)
DR-1	ZnCdSe	0	4.8 ± 0.7	7.2 ± 1.7	27.4 ± 2.9	2.120	172
DR-2	ZnCdSe	10	5.2 ± 0.7	6.2 ± 1.0	28.8 ± 3.0	2.094	126
DR-3	ZnCdSe	60	4.8 ± 0.6	6.0 ± 1.3	25.2 ± 3.6	2.051	67
DR-4	CdSe	0	4.3 ± 0.5	5.4 ± 1.5	21.7 ± 11.6	2.031	34

a Core Composition, S-Injection Delay Time for CdS-Shell Growth (Δ*t*), Average Rod Diameter in the Center (*D*), at the Thickened Side (*C*) and Averaged DR Lengths (*L*), Maximum PL-Emission Energies *E*_PL_ of the Main Peak and Intensity Averaged Fluorescence Lifetimes τ_ave_. Geometry Parameters were Quantified from TEM Images of 200 NCs Per Sample.

Determining the exact material composition of the cores is difficult. In an earlier work, we have estimated the Cd fraction for sample DR-1 to be *x* ≈ 50%.^51^ Additionally, we quantified the Cd fraction via extended X-ray absorption fine structure (EXAFS) spectroscopy (see SI). For these measurements, an additional sample set DR-1′ to DR-3′ was synthesized under the same conditions with slightly smaller ZnSe NCs (diameter of 2.86 nm instead of 3.16 nm). Evaluation of the EXAFS data (see SI) yields values of *x* = 0.63, 0.73, and 0.77 for S-injection delay times of Δ*t* = 0, 10, and 60 s, respectively. Due to the smaller diameter, these values represent an upper limit of the values expected in samples DR-1 to DR-3. Principally, a lower initial value for *x* than 50% as for DR-1 seems to be advantageous in order to investigate a broader range between type-II and type-I behavior. However, it is not trivial to prevent the cation exchange of Zn^2+^ to Cd^2+^, given the less stable bond of Zn–Se of 136 kJ/mol compared to Cd–Se of 310 kJ/mol.^52^ The reduction of the cation exchange by lowering the temperature during the CdS-shell growth was not successful, since the growth of a rod-shaped shell was not possible in our synthesis at a temperature of only 250 °C,^51^ while at temperatures above 240 °C, a pronounced cation exchange is already present.^53,54^ Importantly, we emphasize that the long PL lifetimes measured for DR-1 clearly indicate a pronounced type-II band alignment within the nanostructure.

**Figure 2:**
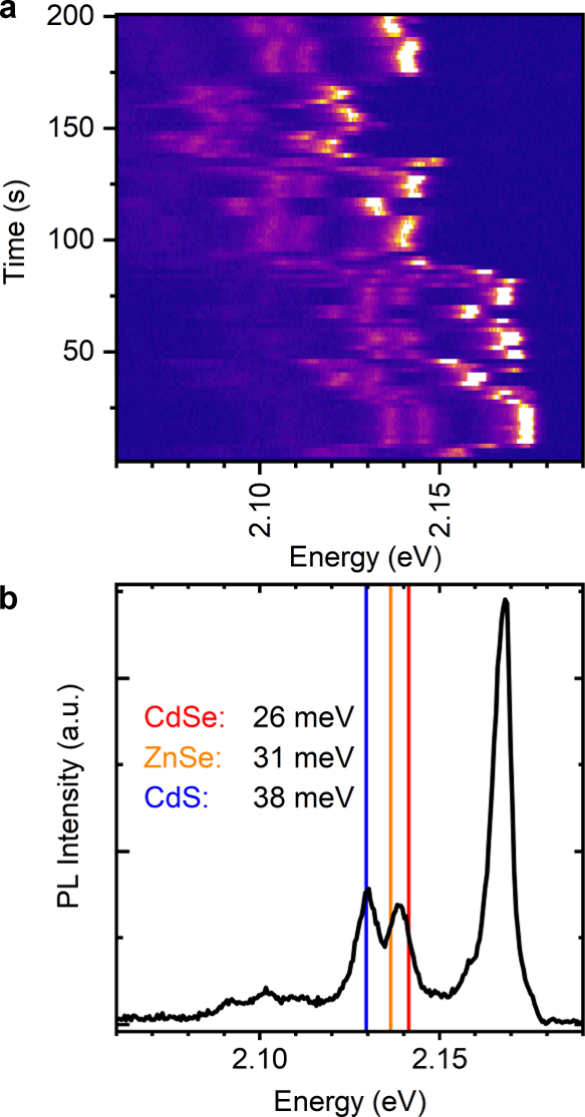
Single-particle spectroscopy at *T* ≈ 10 K. (a) Series of subsequently measured PL spectra of a single NC from sample DR-2, each collected with an integration time of 2 s. (b) PL spectrum of the same DR created by averaging multiple spectra with a similar ZPL energy. The energies of the first phonon lines are highlighted based on bulk values.^55^ The particle was excited at 2.78 eV with a 10 MHz repetition rate.

In the following, we discuss single-nanocrystal PL spectra measured at *T* ≈ 10 K. Figure 2a shows the temporal evolution of the PL spectrum of an exemplary single Zn_1–*x*_Cd_*x*_Se/CdS DR from sample DR-2. The brightest peak corresponds to the zero-phonon line (ZPL), while the energetically lower peaks are phonon replicas arising from exciton–phonon coupling. All of the peaks exhibit collective jittering and spectral jumps in time. This spectral diffusion originates from the formation, movement, and disappearance of surface charges.^56−58^ Spectral diffusion is especially pronounced for a type-II band alignment, since, here, one of the charge carriers is mainly located in the shell, making the exciton more sensitive to the impact of surface charges.

Single-particle spectral time traces were analyzed by averaging those individual spectra within a time trace that exhibit a similar ZPL energy (in the range of 1 nm, i.e., Δ*E* ≈ 2 meV). For the spectral time trace shown in Figure 2a, this results in a spectrum with increased signal-to-noise ratio, as depicted in Figure 2b. Here, the ZPL occurs at *E*_ZPL_ = 2.168 eV. Two LO-phonon replicas can be distinguished as first order phonon lines separated from the ZPL by 28 and 38 meV, respectively. Signatures of higher-order replicas are visible in a broader range around 70 meV below the ZPL energy. For comparison, literature values of the bulk LO-phonon energies^55^ of CdSe, ZnSe, and CdS are plotted as vertical lines relative to *E*_ZPL_. Since the first LO-phonon replica occurs between the references of CdSe and ZnSe, it is called a Se-type replica, with an energy Δ*E*_Se_ below *E*_ZPL_. The second LO-phonon replica, which matches the CdS-phonon reference, is called an S-type replica.

Averaging of the spectra for one ZPL per NC was performed on approximately 100 DRs for each sample. From these particles, 40–70% provided spectra with a sufficient signal-to-noise ratio concerning the LO-phonon replicas. The average Se-type phonon energies are Δ*E*_Se_ = 28 ± 0.7 meV (DR-1), 28 ± 0.5 meV (DR-2), 28 ± 0.7 meV (DR-3), and 27 ± 0.4 meV (DR-4). These values do not indicate a conclusive trend. For a gradual increase in Cd fraction *x* in the Zn_1–*x*_Cd_*x*_Se core, one might expect a continuous decrease of Δ*E*_Se_.^59^ If it exists, this trend seems to be too small to be resolved with respect to the emission-line widths of the presented data. However, consistent with above considerations, DR-4 shows the lowest value of Δ*E*_Se_. (For statistical results, see Table S2) The average S-type phonon energies Δ*E*_S_ are 36 ± 0.4 meV (DR-1), 38 ± 0.3 meV (DR-2), 37 ± 0.7 meV (DR-3) and 36 ± 0.6 meV (DR-4). Here, no significant trend can be deduced, nor would this have been expected.

**Figure 3:**
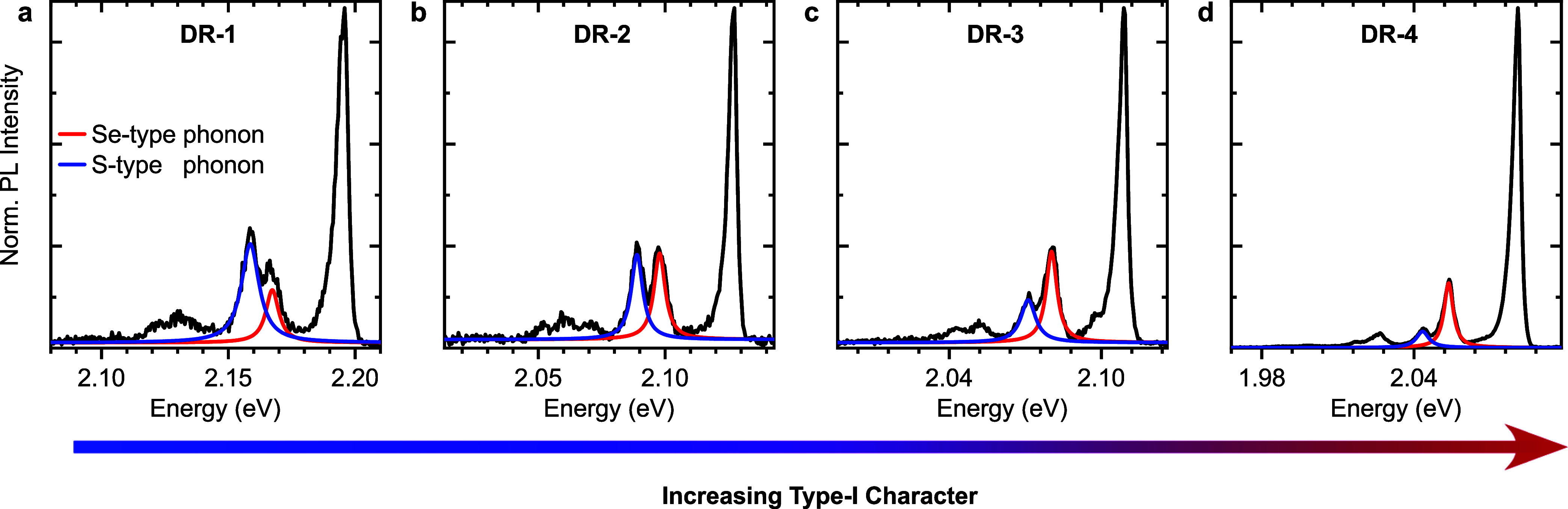
Representative averaged PL spectra of single DRs from (a) sample DR-1 to (d) sample DR-4 measured at *T* ≈ 10 K. The spectra were normalized to their ZPL intensity. The respective first order phonon peaks are highlighted with Lorentzian fits.

Even though the energy of the phonon replicas cannot be correlated to the Cd fraction *x*, a correlation of their relative intensities is possible, as will be shown below for the first-order replicas. Figure 3 displays representative averaged PL spectra of individual particles for each sample, normalized to the ZPL intensity maximum. These spectra suggest that the coupling to LO phonons generally decreases with increasing Cd content in the core, corresponding to an increase in type-I character. The relative intensity of the first order S-type phonon decreases compared to that of the Se-type phonon across the sample series.

**Figure 4:**
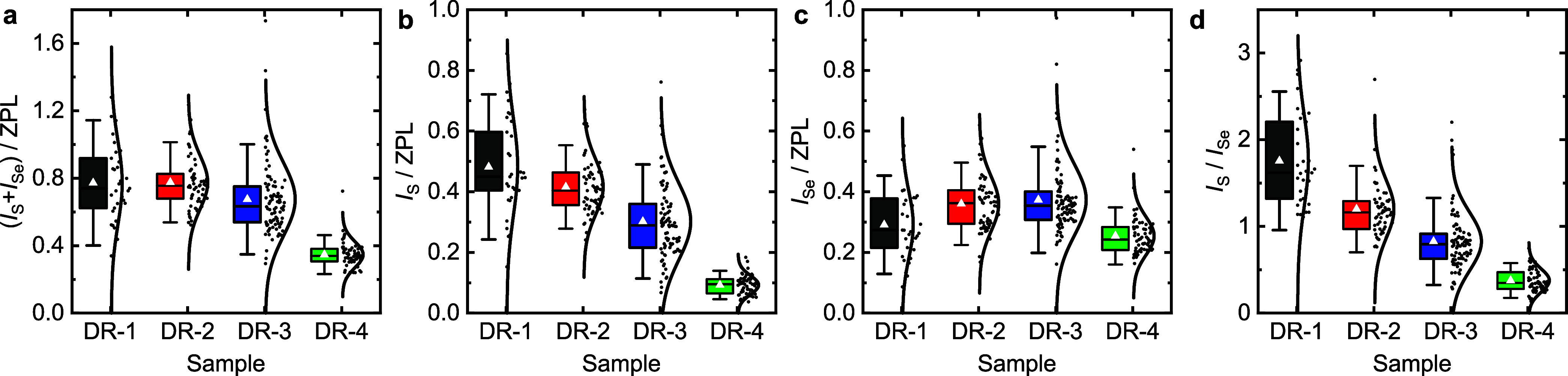
Statistical evaluation of the first order phonon intensities from single DRs. (a–c) Ratios of the sum of (a) S- and Se-type, (b) S-type, and (c) Se-type phonon intensity with respect to the ZPL intensity. (d) Ratio of the S- and Se-type phonon intensities. The boxes cover the interquartile range, while the whiskers show the 95th percentile. The white triangles indicate the average values and the horizontal lines inside the boxes indicate the median values. On the right side of the boxes each black dot corresponds to the phonon ratio measured from a single DR. In the respective samples DR-1 to DR-4 33, 59, 91, and 63 single DRs were evaluated. The curves fit the data points based on normal distributions.

To quantify the phonon coupling, the peaks in each averaged PL spectrum of individual DRs were fitted by Lorentzian functions, and the following combinations of first order and ZPL intensity ratios were evaluated: (*I*_S_ + *I*_Se_)/*I*_ZPL_, *I*_S_/*I*_ZPL_, *I*_Se_/*I*_ZPL_, and *I*_S_/*I*_Se_. Figure 4 summarizes their statistical distributions (for further statistics see Table S2). The intensities are not uniform and scatter to different degrees. The relative total first-order LO-phonon coupling (*I*_S_ + *I*_Se_)/*I*_ZPL_ (Figure 4a) decreases within the sample series, i.e., with increasing type-I character. The relative S-type phonon intensity *I*_S_/*I*_ZPL_ (Figure 4b) gradually decreases with increasing type-I character, while the Se-type phonon intensity *I*_Se_/*I*_ZPL_ (Figure 4c) follows no apparent trend and changes on a smaller scale. The intensity ratio of the S-type to Se-type phonon *I*_S_/*I*_Se_ (Figure 4d), which describes the coupling independent of the ZPL, decreases with an increasing type-I character.

In the following, we qualitatively discuss the behavior of the phonon replica intensities. In ionic semiconductors, the exciton-LO-phonon coupling is primarily facilitated by the Fröhlich interaction,^60^ which describes the coupling of the excitonic charge distribution to the LO-phonon via Coulomb interaction.^61,62^ The decrease of the total LO-phonon coupling apparent in Figure 4a can be explained with a larger charge-carrier overlap for the type-I band alignment to the type-II case.^63,64^ The decrease of the total LO-phonon coupling apparent in Figure 4a can be explained with a larger charge-carrier overlap for the type-I band alignment than for the type-II case.^63,64^ The major change of the S-type phonon (Figure 4b) correlates well with changes of the charge-carrier localization within the sulfide shell of the DRs (Figure 1a–c). As the band alignment changes from type-II to type-I, the probability density of the electron in the shell and thus the phonon coupling to the S-type mode is decreased. The nonmonotonic minor change of the Se-type phonon intensity (Figure 4c) indicates a complex coupling mechanism. The overall lowest Se-type phonon coupling for the type-I system reveals that an overlap of the electron with the hole can in part cancel the Fröhlich coupling.

To compare our observations with the literature, we first refer to a paper by Groeneveld and de Mello Donegá,^43^ which compares the exciton–phonon coupling in type-II bipod-shaped and prolate CdTe/CdSe nanocrystals. The stronger phonon coupling in the bipod-shaped system, as measured by photoluminescence excitation (PLE) spectroscopy, has been attributed to a decreased electron–hole overlap that leads to an enhanced Fröhlich interaction. In these experiments, it was not possible to resolve different phonon replicas of the heterostructure because of the similar LO-phonon energies of CdTe and CdSe. Consequently, only the total LO-phonon coupling was measured.

Second, we note that Gong et al.^65^ investigated the exciton–phonon coupling in spherical Zn_1–*x*_Cd_*x*_Se NCs of varying x by using resonance Raman spectroscopy. These NCs can be regarded as the core-only analogue of our Zn_1–*x*_Cd_*x*_Se/CdS DRs. With increasing *x*, a large decrease in exciton–phonon coupling was observed, which has been explained by charge-carrier localization in locally Cd-rich regions. A corresponding decrease of the Se-type phonon in our case is not observed (cf. Figure 4c) nor expected since our high-temperature cation exchange here has been shown to lead to homogeneous alloying.^53,54^

Third, it has been shown that for spherical CdSe-core/CdS-shell NCs, the exciton–phonon coupling is dependent on the shell thickness. Lin et al.^66^ reported that the shell growth reduced the magnitude of exciton–phonon coupling, in contrast to their simulations. Cui et al.^38^ later suggested that this is only the case for small shell thickness and the reduction of exciton–phonon coupling originates from surface passivation. This, as well as the above-mentioned work on CdTe/CdSe bipod-shaped and prolate NCs, shows that changing the geometry of the nanostructure can affect the exciton–phonon coupling in different ways. In our work, we minimized the influence of the geometry by keeping it nearly constant for our different Zn_1–*x*_Cd_*x*_Se/CdS DRs samples.

Figure 4c shows not only the general trend of the phonon-coupling strengths but also the widespread coupling strengths within each sample. This spread can be a result of the particle-size distribution, different degrees of cation exchange, and varying surface-charge constellations. For reference, Empedocles et al.^56^ found strong deviations of exciton–phonon coupling for single CdSe and CdSe/ZnS spherical NCs, with coupling constants between 0.06 and 1.3 and an average of 0.488. Deviations have also been observed for nominally identical vapor-phase grown InGaAs NCs.^67^

A quantitative modeling of the coupling of excitons to the different LO-phonon modes in heterostructured DRs is a complicated task. Only recently, Lin et al.^45^ modeled the coupling for spherical type-I CdSe/CdS NCs atomistically. A quantitative modeling of anisotropic type-II and type-I heterostructured DRs as investigated here has not yet been accomplished and is beyond the scope of this work.

The exciton–phonon coupling strength is commonly described by the Huang–Rhys factor,^68^ which is given by the intensity ratio of the first order phonon replicas to the ZPL. In heteronanostructures, in particular with a type-II band alignment, this approach has to be modified to account for different couplings in different materials of both electrons and holes. It is well-known that electrons and holes couple differently to phonons.^69^ We propose a simple model in which the coupling is proportional to the probability^63,67,70,71^ of the charges in the shell or the core. For the relative S-type and Se-type phonon replica intensities, we presume
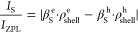
1and
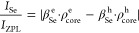
2respectively, with coupling constants β_S,Se_^e,h^ and probabilities
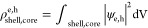
3This approach accounts for the possibility that overlapping electron and hole wave functions ψ_e,h_ can suppress the overall phonon coupling.

**Figure 5:**
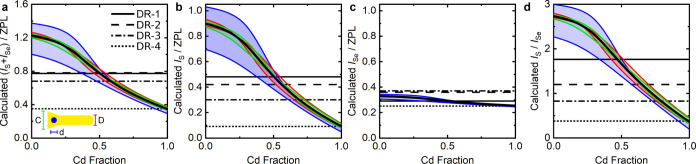
Estimation of the properties depicted in Figure 4 is based on theoretical calculations within the effective mass approximation. The following phonon ratios are illustrated: (a) (*I*_S_ + *I*_Se_)/*I*_ZPL_, (b) *I*_S_/*I*_ZPL_, (c) *I*_Se_/*I*_ZPL_, and (d) *I*_S_/*I*_Se_. The black curves were calculated using the average geometry of the samples. The colored lines were calculated by independently changing the core (*d* = 3.17 ± 0.43 nm), rod (*D* = 4.78 ± 0.62 nm), and cone (*C* = 6.21 ± 1.40 nm) diameters according to their averaged standard deviations obtained from TEM data and keeping the rod length at 25.78 nm. The inset depicts the schematic cross-section that was used in the calculations. The horizontal lines indicate the average experimental values.

The probabilities were calculated within the effective mass approximation.^72,73^ Details on the calculations can be found in the Supporting Information. The choice of material parameters used for the calculations, in particular, the different band-edge energies and effective masses, are also discussed in the SI; they are listed in Table S3. Note that we neglected strain effects in our model calculations, which are expected to be small and to not significantly change the band alignment.^74^ The geometry of the DRs was approximated by a spherical core (diameter *d*), embedded in a rod-shaped shell (diameter *D*, length *L*). The core was placed on one end of the rod such that it was covered by 3 monolayers of shell material. The bulge-like shape of the DRs is modeled as a truncated cone (base diameter *C*, height *C*/2, cutting-surface diameter *D*; see inset of Figure 5). Average geometry parameters that represent samples DR-1 to DR-4 were determined from spectroscopy of the core sample (*d*) and from TEM investigations (*D*, *L*, and *C*). For the Zn_1–*x*_Cd_*x*_Se-core material, a homogeneous cation distribution was assumed.^51,53,54,75^

As mentioned above, the Cd content *x* in the Zn_1–*x*_Cd_*x*_Se-core of samples DR-1 to DR-3 is subject to some uncertainties. It is best known for sample DR-1, for which we assume *x* = 0.50.^51^ For sample DR-4, *x* = 1 is valid. With that, we calculated the ρ_shell,core_^e,h^ for samples DR-1 and DR-4. Together with the experimentally determined values of the ratios *I*_S,Se_/*I*_ZPL_ for both samples, eqs 1 and 2 represent a linear system of equations with the four unknowns β_S,Se_^e,h^. Solving this system of equations yields the values β_S_^e^ = 1.35, β_S_^h^ = 3.35, β_Se_^e^ = 0.12, and β_Se_^h^ = 0.38. Interestingly, these values exhibit similarities to the bulk Fröhlich-coupling constants α (see Table S4). Like the corresponding Fröhlich constants, the coupling constants β are larger for the hole than for the electron, and they are larger for CdS than for ZnSe and CdSe. Furthermore, the ratio β_S_^e^/β_S_^h^ is similar to that of α_S_^e^/α_S_^h^.

The coupling constants β_S,Se_^e,h^ have then been used—together with results from COMSOL simulations—to estimate the relative phonon-coupling strengths. Results are given in Figures 5, where panels b and c represent relative S- and Se-type phonon intensities calculated by eqs 1 and 2, respectively, while the consequential ratios (*I*_S_ + *I*_Se_)/*I*_ZPL_ and *I*_S_/*I*_Se_ are depicted in panels a and d, respectively, analogous to Figure 4.

In each panel, the black curve is obtained for geometry averaged over all four samples. The horizontal lines represent the experimental average values for DR-1–4. The modeling demonstrates that the coupling strength to the S-type phonon is strongly decreasing with increasing *x* (see Figure 5b). This is in line with the experimentally observed decrease from about 0.5 for *x* = 0.5 (DR-1) to 0.1 for *x* = 1 (DR-4). The modeling results estimate the Cd fraction of samples DR-2 and DR-3 to 0.57 and 0.69, respectively. The modeled coupling to the Se-type phonon is essentially constant (between 0.35 and 0.25 for 0 < *x* < 1, see Figure 5c). A subtle increase in the coupling for DR-2 and DR-3 observed in the experiment cannot be reproduced by the model. The modeled total coupling strength in Figure 5a decreases with increasing *x*; it is dominated by the coupling to the S-type phonon. Figure 5d shows the coupling ratio of the S- to Se-type phonon intensity, independently of the ZPL. Using these data to again estimate *x* for samples DR-2 and DR-3 yields values around 0.65 and 0.79, respectively. These values are on a similar scale as the values determined by EXAFS spectroscopy.

Numerical modeling allows us to investigate the influence of each geometry parameter on the intensity ratios. For this, the calculations were repeated and the diameters of the rod *D*, cone-base *C*, or core *d* were separately increased or decreased by their experimental standard deviation. Results are given by the colored curves in Figure 5. It can be deduced that the spread of different core diameters within a sample has the strongest effect, while the cone and rod diameters have a weaker effect. The position of the core and different band offsets have a neglectable influence (see Figure S8). Considering the variation of all geometry parameters together, one would expect a broader distribution of phonon-coupling ratios for small *x*. This agrees with the experimentally observed distributions of the data points in Figure 4.

A closer look at the data of Figure 4d reveals that the standard deviation for sample DR-1 is a factor of 4 larger than for DR-4. From the calculated data, summing the deviations for all geometric variations, one would expect the broadening for *x* = 0.5 to be only a factor of 1.5 larger than that for *x* = 1. Therefore an additional contribution might be relevant. It has been reported that surface charges and point defects strongly increases the exciton–phonon coupling in NCs, compared to the intrinsic coupling.^38,41,63,71,76,77^ In order to estimate the influence of surface charges in our system, we placed a positive or negative point charge on the model structure at the position where the conical shape changes into the cylindrical shape (cf. the inset of Figure 5a). The calculated data shown in Figure S9 reveal a strong change of the S-type phonon coupling, which is maximal for *x* ≈ 0.5. Thus, surface charges can explain the experimentally observed broader distribution of S- to Se-type phonon ratios for DR-1. In particular, negative surface charges strongly increase the S-type phonon coupling for *x* around 0.5. These charges might cause the experimentally determined averages of the relative S-type phonon intensities to be larger than their median values, as depicted in Figure 4b and d.

## Conclusions

In conclusion, we investigated charge-carrier localization in Zn_1–*x*_Cd_*x*_Se/CdS DRs with different compositions. Optical and X-ray spectroscopy proved that in situ cation exchange is suitable for band engineering, offering the possibility to continuously tune charge-carrier localization and energy. This allowed us to design a set of samples with different band alignments, while keeping the geometry comparable. Statistical data obtained from cryogenic-temperature spectroscopy on the single-particle level revealed that the first-order LO-phonon ratio of the S- to Se-type phonon replica changes within the set of samples and is thus an indicator of different charge-carrier localization. Our simplified theoretical model for the coupling ratios captures the general trend of the phonon ratios. Our study shows that exciton–phonon coupling can be used as a new quantity characterizing the exciton localization. Atomistic calculations for exciton–phonon coupling are still actively under development.^45,78−80^ Our experimental statistical data at the single-particle level can serve as the basis for future comparisons with new atomistic simulations, so that the present discrepancies between experiment and theory will be resolved.

## Supporting Information Available

Description of the synthesis; TEM data; UV/vis spectra; EXAFS analysis; single-particle statistics from PL measurements and calculation details (PDF)
