# Supplementary material for: Exciton–Phonon Coupling in Single ZnCdSe-Dot/CdS-Rod Nanocrystals with Engineered Band Gaps from Type-II to Type-I
Source: ACS Photonics. 2024 Sep 6;11(9):3741–9. doi: 10.1021/acsphotonics.4c00931 (PMC11413927; doi:10.1021/acsphotonics.4c00931)
Supplement: Supplementary file 1 — ph4c00931_si_001.pdf [file ph4c00931_si_001.pdf]

Supporting Information for

Exciton–Phonon Coupling in Single ZnCdSe-Dot/CdS-Rod Nanocrystals with Engineered Band Gaps from Type-II to Type-I

Florian Johst,† Jannik Rebmann,† Hans Werners,† Lars Klemeyer,‡ Jagadesh Kopula Kesavan,‡ Dorota Koziej,‡ Christian Strelow,† Gabriel Bester,†,¶ Alf Mews,† and Tobias Kipp*,†

†*Institute of Physical Chemistry, University of Hamburg, Grindelallee 117, D-20416 Hamburg, Germany*

‡*Institute of Nanostructure and Solid State Physics, University of Hamburg, Luruper Chaussee 149, D-22761 Hamburg, Germany*

¶*The Hamburg Centre for Ultrafast Imaging, Luruper Chaussee 149, Hamburg 22761, Germany*

E-mail: tobias.kipp@uni-hamburg.de

Chemicals

Acetone (≥ 99.5 %, Th. Geyer / 99.8 %, VWR Chemicals), cadmium oxide (99.9999 %, ChemPur), cadmium myristate ($Cd(myr)_2$, synthesized by Julia Funk from the group of Horst Weller), cadmium nitrate tetrahydrate ($Cd(NO_3)_2$, 99.997 %, Merck), chloroform (99 %, VWR Chemicals), diethylzinc in hexane (Sigma-Aldrich), *n*-hexane (99.7 %, VWR Chemicals), hexylphosphonic acid (HPA, >99 %, PCI Synthesis), methanol (99.5 %, Grüssing), 1-octadecen (ODE, ≥ 89.5 %, Sigma-Aldrich), octadecylamine (ODA, ≥ 99 %, Sigma-Aldrich), octadecylphosphonicacid (ODPA, >99 %, PCI Synthesis), oleic acid (OA, >89.5 %, Sigma-Aldrich), oleylamine (OAm, 70 %, Sigma-Aldrich), 2-propanol (99.7 %, VWR Chemicals), selenium (Se, 99.5 %, Acros Organics), sodium myristate (>98 %, TCI Chemicals), sulphur (S, 99.8 %, Sigma-Aldrich), toluene (>99.5 %, VWR Chemicals / 99.85 %, Acros Organics), tri-octylphosphine (TOP, 97 % ABCR), tri-octylphosphine oxide (TOPO, 99 %, Sigma-Aldrich). Chemicals were used without further purification.

Synthesis

The ZnSe/CdS synthesis follows the same procedure as described in,[1] which was adapted from the works of Cozzoli et al.,[2] Carbone et al.,[3] Dorfs et al.[4] and Huang et al.[5]

ZnSe dot synthesis: 7 g of ODA was degassed at 130 °C under the vacuum and subsequently heated up to 300 °C under the N_2 atmosphere. A mixture of 0.8 mL of a 1 M diethylzinc hexane solution and 2.5 mL of a 0.33 M Se:TOP hexane solution was added, subsequently the temperature was lowered to 265 °C. After a reaction time of 30 min, 1 mL of the aforementioned Zn/Se mixture was added dropwise over a period of 5 min, this addition was repeated 6 times. The ZnSe dots were isolated by extraction with toluene and methanol, followed by centrifugation at 40 °C. The ZnSe dots were mixed with TOP. The average ZnSe dot diameter was determined from its UV/Vis absorption.[6]

CdSe dot synthesis: CdSe dots with a zinc blende phase were synthesized using $Cd(myr)_2$, that was obtained via ion exchange of $Cd(NO_3)_2$ and Na(myr) in methanol at room temper-

ature. 170 mg $Cd(myr)_2$ in 37 mL ODE was degassed under vacuum at 10 °C. Afterwards, 33 mg Se was added under the N_2 atmosphere and the mixture was heated up to 240 °C. 1.0 mL OA and 4.0 mL OAm were added to the mixture. The reaction was quickly cooled down after 4 min, followed by the addition of acetone. The mixture was centrifuged for 10 min at 11 000 rpm. The precipitate was dissolved in toluene and precipitated by addition of methanol. After a second centrifugation step, the precipitate was re-dispersed in TOP. The average CdSe dot diameter was calculated from the UV/Vis absorption.[7]

ZnCdSe/CdS DR-1 synthesis: 60 mg CdO, 290 mg ODPA, 80 mg HPA and 3 g TOPO were degassed under the vacuum at for 90 min at 150 °C. After the solution was heated to 320 °C 1.8 mL of a 2 M S:TOP solution containing 40 nmol ZnSe dots was injected. The reaction was stopped after 8 min by quickly cooling down. The DRs were isolated by adding a mixture of toluene and methanol, followed by centrifugation for 10 min at 11 000 rpm. This cleaning step was repeated three times. The isolated DRs were re-dispersed in toluene.

ZnCdSe/CdS DR-2 and DR-3 synthesis: The synthesis was performed similar to DR-1. Instead of injecting the Se and S precursors simultaneously, first 40 nmol ZnSe dots in 0.5 mL TOP were injected. After a delay of 10 s for DR-2 and 60 s for DR-3 1.8 mL of a 2M S:TOP solution was injected.

CdSe/CdS DR synthesis: The CdSe/CdS DRs synthesis is identical to that of the ZnSe/CdS DRs, except CdSe dots were used as seeds instead of ZnSe dots.

Transmission Electron Microscopy

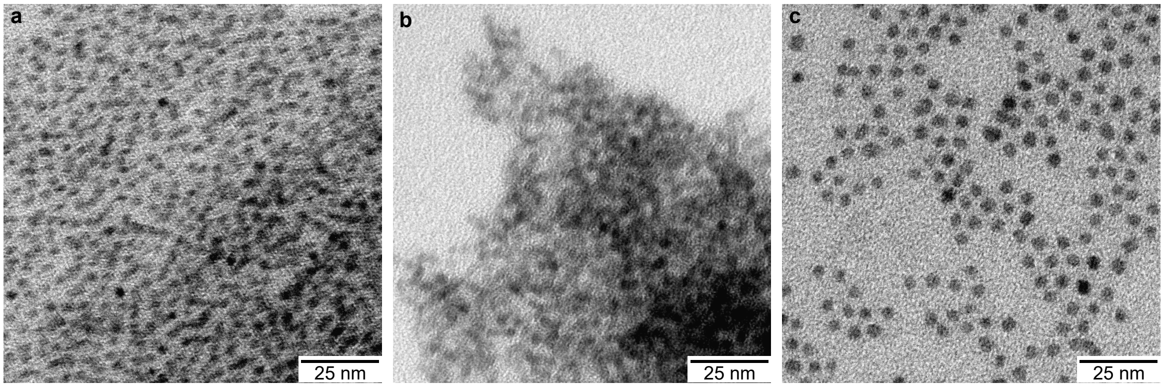

Figure S1: TEM images of (a,b) ZnSe dots and (c) CdSe dots with average diameters $d = 3.16$ nm, $d = 2.78$ nm and $d = 3.18$ nm respectively.

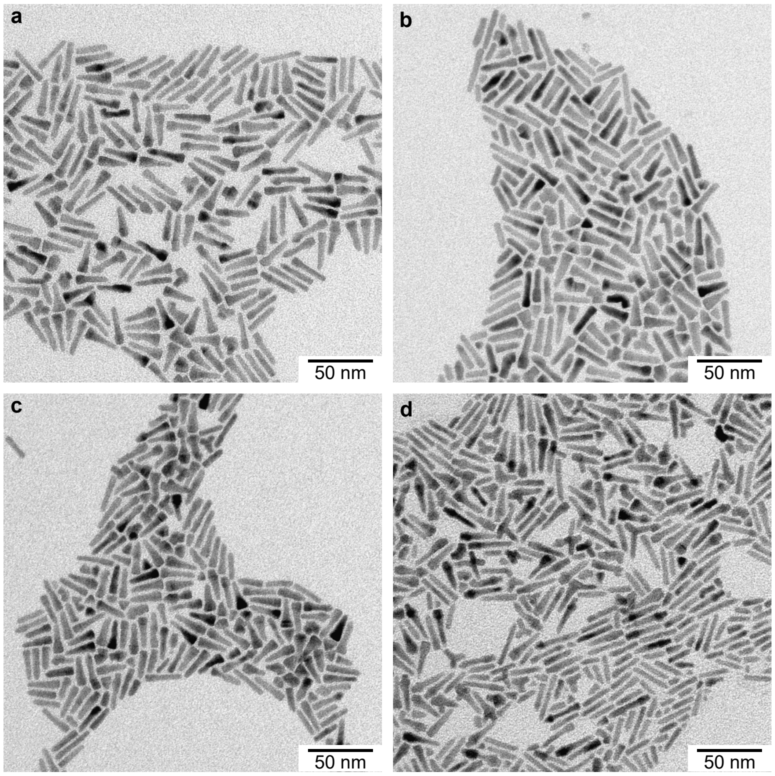

Figure S2: TEM images of the sample (a–d) DR-1–DR-4

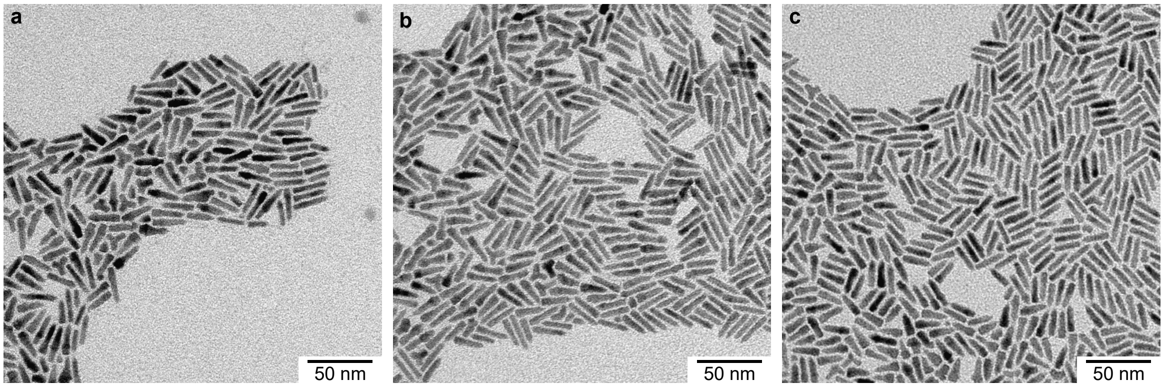

Figure S3: TEM images of the samples (a–c) DR-1′–DR-3′.

Transmission electron microscopy (TEM) images were acquired with a JEM-1011 (JEOL) with an acceleration voltage of 100 kV.

UV/Vis Absorption and Emission in Solution

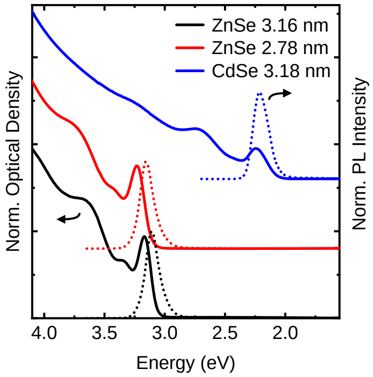

Figure S4: Ensemble absorption (solid lines) and photoluminescence spectra (dotted lines) of ZnSe and CdSe cores measured at room temperature.

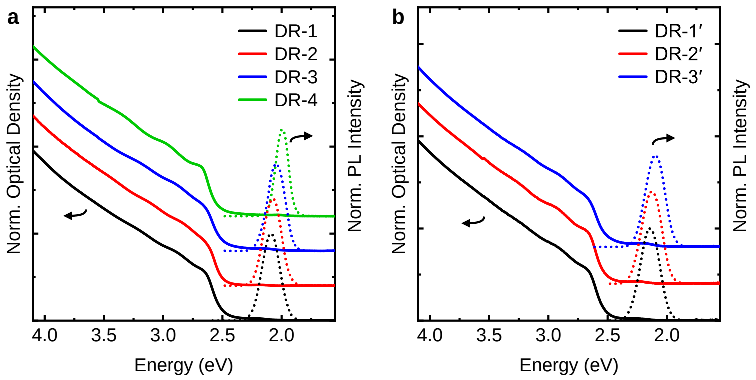

Figure S5: Ensemble absorption (solid lines) and photoluminescence spectra (dotted lines) of (a) the samples DR-1–DR-4 and (b) the samples DR-1′–DR-3′ set measured at room temperature.

Absorption and emission spectra were measured using the commercial spectrometer Cary 5000 UV-Vis-NIR (Varian). All samples were diluted in toluene and measured in quartz cuvettes with a path length of 1 cm.

X-ray Absorption Spectroscopy

For the X-ray absorption spectroscopy all samples were dried and diluted with cellulose. The data was measured on the Se K-edge (12657.8 eV) at room temperature in fluorescence mode at the P64[8] beamline of PETRA-III at the German Electron Synchrotron (DESY) in Hamburg. A Se-foil was used as a reference to ensure the energy calibration shifts less than 0.1 eV. Spectral data were processed using ATHENA[9] with the software package IFEFFIT. Scattering paths for the evaluation of the extended X-ray absorption fine structure (EXAFS) data were calculated with the software package FEFF 6.0 using ARTEMIS.[9] The Fourier transformed (FT) spectra were fitted in the range of 3–15 Åwith a K^3 weight, and the backward FT spectra in the range of 1.5–4.7 Å. The R-factor was below 0.02 for all fits. The many-body amplitude factor ($S_0{}^2$) was fixed to 0.88, as determined from the Se-foil. The following fitting parameters were used: the interatomic distances, the energy origin shift and Debye–Waller factors. The multi-shell fitting method used here is based on physical constraints given by the crystallographic structure.[10,11] For the respective scattering path

contributions both ZnSe and CdSe were assumed to be in the zinc blende phase.

Figure S6a shows the X-ray absorption near-edge structure (XANES) of DR-1′–DR-3′, the ZnSe cores used in their synthesis and DR-4. The spectral features in the XANES of ZnSe core and $Zn_{1-x}Cd_xSe$ alloyed samples are shifting of 0.4 eV towards lower energy, which suggest an increase of Cd content in the vicinity of Se scatterer. The FT EXAFS spectra are depicted in Figure S6b. The evaluated Cd fraction, coordination numbers and interatomic distances are listed in Table S1. The EXAFS data reveal that without promotion of the cation exchange during the reaction, the Cd content in the core increases strongly. Furthermore, the injection delay of the S-precursor resulted in a Cd increase. This is consistent with the decreasing Zn and increasing Cd coordination numbers. The total coordination number for the first shell of the Se varies around 3.5. Most noticeably, the second shell coordination to Se decreases for the $Zn_{1-x}Cd_xSe$ alloys compared to the pure systems.

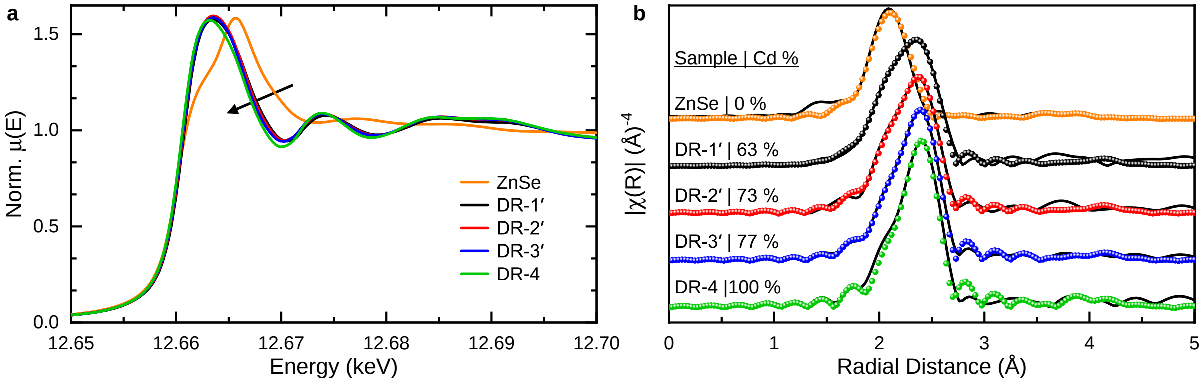

Figure S6: X-ray absorption spectroscopy (XAS) measurements at Se Ka-edge of ZnSe cores (orange), DR-1′ (black), DR-2′ (red), DR-3′ (blue) and DR-4 (green); (a) the normalized XANES spectra and (b) Fourier Transformed EXAFS spectra. The solid, dotted lines correspond to the measured data and calculated fits respectively. The black arrow points at the energy shift in the XANES spectra.

Table S1: Results of the EXAFS evaluation. Listed are the Cd contents, coordination numbers N_{Zn}, N_{Cd}, N_{Se} and interatomic distances $R_{\text{Se-Zn}}$, $R_{\text{Se-Cd}}$.

Sample	Cd (%)	N_{Zn}	N_{Cd}	N_{Se}	$R_{\text{Se-Zn}}$ (Å)	$R_{\text{Se-Cd}}$ (Å)
ZnSe	0.00	3.38 ± 0.12		10.68 ± 5.11	2.420 ±0.01	
DR-1′	63.19	1.32 ± 0.16	2.23 ± 0.16	7.50 ± 3.63	2.448 ± 0.01	2.627 ± 0.01
DR-2′	72.50	0.92 ± 0.16	2.58 ± 0.15	6.73 ± 3.74	2.451 ± 0.01	2.618 ± 0.01
DR-3′	76.80	0.58 ± 0.13	2.95 ± 0.12	7.00 ± 4.21	2.445 ± 0.01	2.616 ± 0.01
DR-4	100.00		3.66 ± 0.20	10.16 ± 4.80		2.603 ± 0.01

Low-Temperature Spectroscopy

For low-temperature spectroscopy all samples were diluted in *n*-hexane. The diluted samples were mixed with a Vortexer for 60 s and then exposed to ultrasonic in order to dissolve any agglomerates within the colloidal solutions. After this treatment 10 µL of each sample was spin-coated at 5000 rpm for 15 s onto a 5x5 mm Si wafer with a thermally grown 300 nm SiO_2 layer. Beforehand the wafers were cleaned sequentially with acetone, 2-propanol and water in an ultrasonic bath for 20 min each. The sample dilution was adjusted for ensemble and single particle measurements accordingly.

The measurements were performed using a home built confocal laser scanning microscope. Particles were excited at 446 nm using a PiL044X laser diode (Advanced Laser Diode Systems) with a EIG2000DX controller (Advanced Laser Diode Systems). For ensemble PL-spectra the laser was set to continuous wave operation with an intensity of 50 nW, while pulsed excitation with a repetition rate of 1 MHz and laser intensity of 2 nW was used for time-resolved PL ensemble measurements. Single particle PL spectra were measured with a repetition rate of 10 MHz and a laser intensity of ~80 nW.

The laser light was filtered with a 450 nm short-pass filter. For ensemble measurements the light was collected with a $f = 100$ mm lens and for single particle measurements a LM-PLFLN 100X objective (N.A. of 0.8, Olympus) was used. The laser light was blocked in the detection beam path by a 488 nm long pass filter. The time-resolved PL was measured with a PDM Series Free Space (Micro Photon Devices) avalanche photodiode and a PicoHarp300

(PicoQuant) time-correlated-single-photon-counting device. PL spectra were collected with an Acton SP-2-500i (Princeton Instruments) spectrograph and a PIXIS 400B (Princeton Instruments) charge-coupled device camera. The samples were cooled down to ~10 K using a customized attoDRY 700 (Attocube) closed-cycle He-cryostat.

Table S2: Statistical data for single particle spectroscopy of DR-1–DR-4 at around 10 K. Listed are the zero-phonon line energies E_{ZPL}, the first order phonon energies $E_{\mathrm{LO_1(Se/S)}}$ and different intensity ratios for the first order phonon replica and zero phonon line intensities $I_{\mathrm{LO_1/ZPL}}$.

	DR-1	DR-2	DR-3	DR-4
E_{ZPL} (eV)	2.180 ± 0.052	2.154 ± 0.051	2.111 ± 0.051	2.082 ± 0.030
$E_{\mathrm{LO_1(Se)}}$ (meV)	28 ± 0.7	28 ± 0.5	28 ± 0.7	27 ± 0.4
$E_{\mathrm{LO_1(S)}}$(meV)	36 ± 0.4	38 ± 0.3	37 ± 0.7	36 ± 0.6
$I_{\mathrm{LO_1(S)}}/I_{\mathrm{LO_1(Se)}}$	1.76 ± 0.53	1.20 ± 0.33	0.83 ± 0.33	0.38 ± 0.13
$(I_{(\mathrm{LO_1(Se)}}+I_{\mathrm{LO_1(S)})})/I_{\mathrm{ZPL}}$	0.77 ± 0.24	0.78 ± 0.16	0.69 ± 0.24	0.35 ± 0.08
$I_{\mathrm{LO_1(Se)}}/I_{\mathrm{ZPL}}$	0.29 ± 0.11	0.36 ± 0.09	0.37 ± 0.12	0.25 ± 0.06
$I_{\mathrm{LO_1(S)}}/I_{\mathrm{ZPL}}$	0.48 ± 0.16	0.42 ± 0.09	0.30 ± 0.13	0.09 ± 0.03

Calculations

To calculate the electron and hole wave functions, we solved the Schrödinger and Poisson equations iteratively, as described by Panfil et al.[12]

The equations were solved numerically in COMSOL Multiphysics 5.4 using tetrahedral meshes with approximately 150 000 mesh elements, which varied slightly depending on the calculated sample geometry. The Dirichlet boundary condition was used to set the wave function to zero 15 nm away from the particle surface, while the von Neumann boundary condition is used for all inner domain boundaries. The electron and hole wave functions $\Psi_{\mathrm{e/h}}$ are calculated by solving the Schrödinger equation

$$\Big(-\frac{\hbar^2}{2m_{\mathrm{e/h}}}\nabla^2 + V_{\mathrm{vb/cb}}\Big)\Psi_{\mathrm{e/h}} = E\Psi_{\mathrm{e/h}}, \tag{1}$$

with the respective effective masses $m_{\mathrm{e/h}}$ and band potentials $V_{\mathrm{vb/cb}}$. The resulting proba-

bility densities $|\Psi_{\mathrm{e/h}}|^2$ are then used in the Poisson equation

$$-\nabla(\epsilon_0\epsilon_{\mathrm{r}}\nabla\phi_{\mathrm{e/h}}) = q_{\mathrm{e/h}}|\Psi_{\mathrm{e/h}}|^2, \tag{2}$$

to derive the electric potentials $\phi_{\mathrm{e/h}}$. The calculated potentials are then introduced in the Hamiltonian and the Schrödinger equations

$$\Big(-\frac{\hbar^2}{2m_{\mathrm{e/h}}}\nabla^2 + V_{\mathrm{vb/cb}} + q_{\mathrm{e/h}}\phi_{\mathrm{h/e}}\Big)\Psi_{\mathrm{e/h}} = E_{\mathrm{e/h}}^{\phi_{\mathrm{h/e}}} \tag{3}$$

that include the Coulomb interaction are solved. Followed by the derivation of new potentials. This process is repeated until the Eigenvalues are converged. After the calculations are converged the exciton energy E_{exc} is calculated as

$$E_{\mathrm{exc}} = E_{\mathrm{e}} + E_{\mathrm{h}} - \frac{E_{\mathrm{e}} - E_{\mathrm{e}}^{\phi_{\mathrm{h}}} + (E_{\mathrm{h}} - E_{\mathrm{h}}^{\phi_{\mathrm{e}}})}{2}. \tag{4}$$

The parameters used for the calculations are listed in Table S3, together with their corresponding references. We have used values from as few different references as possible to avoid picking parameters from different references with a certain bias. The band edge energies for the different material compositions determine whether a heterostructure is type-I or type-II. We assumed the CdSe conduction band to be 0.26 eV below the CdS conduction band, leading to a type-I band alignment. The conduction band of ZnSe was assumed to be 0.71 eV above the CdS conduction band. The conduction band offset for Zn1-xCdxSe was calculated based on the aforementioned offsets and the bowing-term corrected band gap, using the bowing coefficient of 0.387 eV.13 The valence band offset for $Zn_{1-x}Cd_xSe$ was approximated by linear interpolation between the values of ZnSe and CdSe, which are given in Table S3. Figure S7 sketches the band edge energies for ZnSe, $Zn_{0.5}Cd_{0.5}Se$, CdSe and CdS as assumed in this work.

The valence band offset for $Zn_{1-x}Cd_xSe$ was approximated by linear interpolation between

ZnSe and CdSe, while the conduction band offset was calculated based on the aforementioned offset and the bowing-term corrected band gap, using the bowing coefficient of 0.387 eV.[13] All remaining properties of $Zn_{1-x}Cd_xSe$ were interpolated linearly. The CdS monolayer thickness used to define the core position was 0.35 nm.[14]

Table S3: Compilation of the parameters used in the calculations: effective masses $m_{e/h}$, static and high frequency dielectric constants $\epsilon_{0/\infty}$, band gap energies E_g, conduction band offset V_e and valence band offset V_h. The anisotropic properties of CdS in the wurtzite phase are noted orthogonal and parallel to the crystallographic c-axis.

	CdS⊥/∥	ZnSe	CdSe	Vacuum	Ref.
$m_e(m_0)$	0.150 / 0.152	1.00	0.137	0.119	15
$m_h(m_0)$	0.40 / 1.82	0.52	0.33	1.00	15
$\epsilon_s(\epsilon_0)$	10.2 / 9.0	8.9	9.6	1.0	15
$\epsilon_\infty(\epsilon_0)$	5.4 / 5.3	5.9	6.2	1.0	15
E_g(eV)	2.45	2.81	1.77	-	15
V_e(eV)	0.26	0.97	0.00	-	16
V_h(eV)	−0.42	−0.07	0.00	-	16

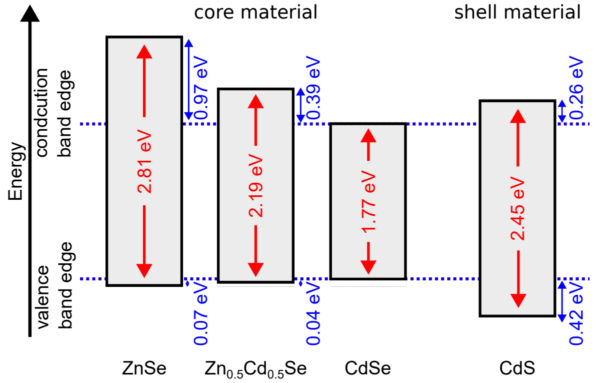

Figure S7: Overview of the valence and conduction band energies of ZnSe, $Zn_{0.5}Cd_{0.5}Se$, CdSe and CdS, based on the references listed in Table S3 and the aforementioned bowing parameter of 0.387 eV.[13] The band gaps are noted in red and the offsets in relation to CdSe in blue.

The Fröhlich coupling constants for the bulk materials were calculated with[17]

$$\alpha_{\mathrm{e,h}} = \frac{e^2}{8\pi\epsilon_0\hbar\omega_{\mathrm{LO}}}\left(\frac{2m_{\mathrm{e,h}}\omega_{\mathrm{LO}}}{\hbar}\right)^{1/2}\left(\frac{1}{\epsilon_\infty}-\frac{1}{\epsilon_s}\right), \quad (5)$$

using the bulk phonon energies of: CdSe = 26 meV, ZnSe = 31 meV, CdS = 38 meV.[18]

Table S4: Fröhlich coupling constants for bulk ZnSe, CdSe and CdS.

	CdS	ZnSe	CdSe
α_{e}	0.62	0.48	0.45
α_{h}	1.49	0.86	0.75

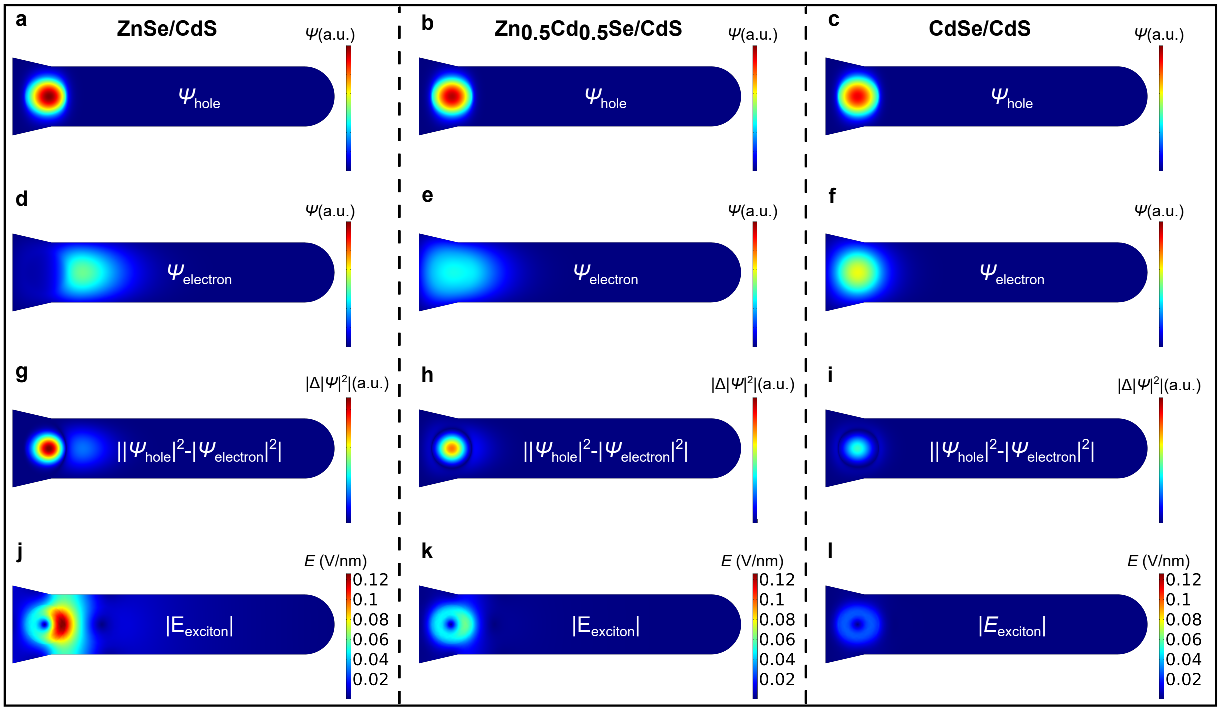

Figure S8: Cross sections of calculated dot-in-rod NCs with different Cd fractions in the core. (a–c) hole wavefunctions, (d–f) electron wavefunctions, (g–i) absolute differences of the probability densities of hole and electron and (j–l) electric field of the exciton.

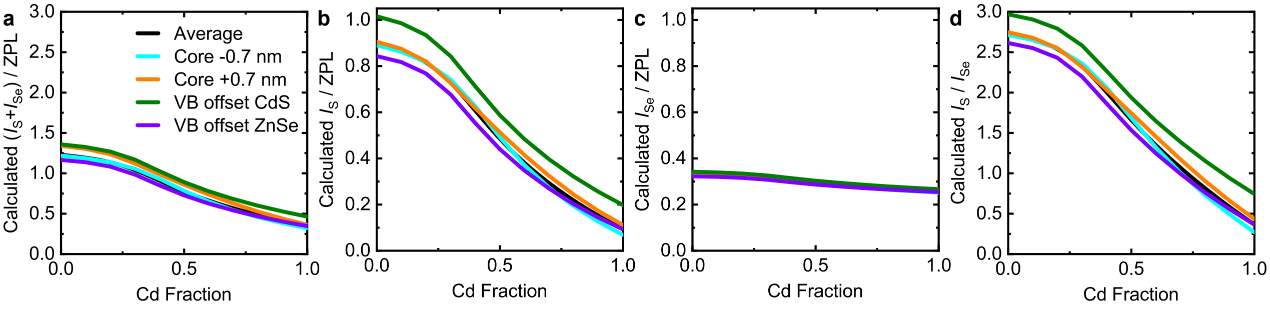

Figure S9: Modified calculations for (a) $(I_{\rm S}+I_{\rm Se})/I_{\rm ZPL}$, (b) $I_{\rm S}/I_{\rm ZPL}$, (c) $I_{\rm Se}/I_{\rm ZPL}$ and (d) $I_{\rm S}/I_{\rm Se}$. Variation of the core position along the *c*-axis towards $[00\bar{1}]$ (+0.7 nm) and away from it (−0.7 nm), as well as larger valence-band offsets for CdS-CdSe 0.55 eV[19] and ZnSe-CdS 0.11 eV.[19]

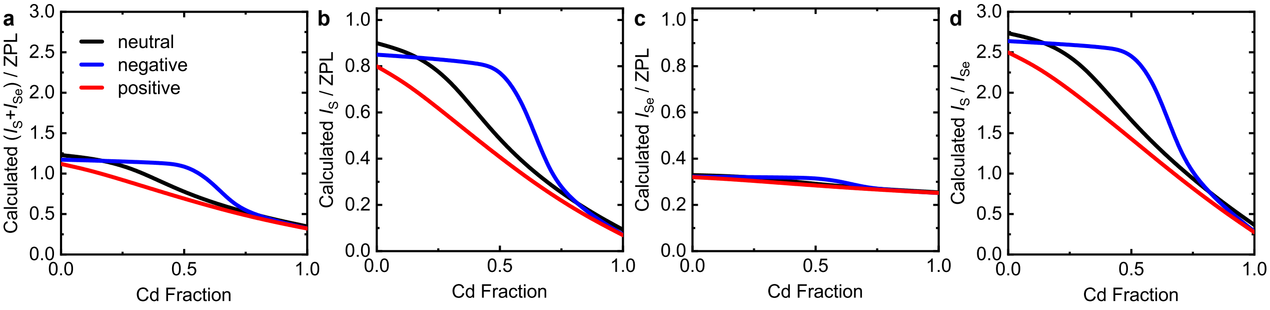

Figure S10: Illustration of the effect of a single point charge on the rod surface at the upper end of the cone. (a) $(I_{\rm S}+I_{\rm Se})/I_{\rm ZPL}$, (b) $I_{\rm S}/I_{\rm ZPL}$, (c) $I_{\rm Se}/I_{\rm ZPL}$ and (d) $I_{\rm S}/I_{\rm Se}$.

References

(1) Rebmann, J.; Werners, H.; Johst, F.; Dohrmann, M.; Staechelin, Y. U.; Strelow, C.; Mews, A.; Kipp, T. Cation Exchange during the Synthesis of Colloidal Type-II ZnSe-Dot/CdS-Rod Nanocrystals. *Chem. Mater.* **2023**, *35*, 1238–1248, DOI: `10.1021/acs.chemmater.2c03278`.

(2) Cozzoli, P. D.; Manna, L.; Curri, M. L.; Kudera, S.; Giannini, C.; Striccoli, M.; Agostiano, A. Shape and Phase Control of Colloidal ZnSe Nanocrystals. *Chem. Mater.* **2005**, *17*, 1296–1306, DOI: `10.1021/cm047874v`.

(3) Carbone, L. et al. Synthesis and micrometer-scale assembly of colloidal CdSe/CdS

nanorods prepared by a seeded growth approach. *Nano Lett.* **2007**, *7*, 2942–2950, DOI: 10.1021/nl0717661.

(4) Dorfs, D.; Salant, A.; Popov, I.; Banin, U. ZnSe quantum dots within CdS nanorods: a seeded-growth type-II system. *Small* **2008**, *4*, 1319–1323, DOI: 10.1002/smll.200800084.

(5) Huang, J.; Kovalenko, M. V.; Talapin, D. V. Alkyl chains of surface ligands affect polytypism of cdse nanocrystals and play an important role in the synthesis of anisotropic nanoheterostructures. *J. Am. Chem. Soc.* **2010**, *132*, 15866–15868, DOI: 10.1021/ja105132u.

(6) Toufanian, R.; Zhong, X.; Kays, J. C.; Saeboe, A. M.; Dennis, A. M. Correlating ZnSe Quantum Dot Absorption with Particle Size and Concentration. *Chem. Mater.* **2021**, *33*, 7527–7536, DOI: 10.1021/acs.chemmater.1c02501.

(7) Manthiram, K.; Beberwyck, B. J.; Talapin, D. V.; Alivisatos, A. P. Seeded synthesis of CdSe/CdS rod and tetrapod nanocrystals. *J. Vis. Exp.* **2013**, e50731, DOI: 10.3791/50731.

(8) Caliebe, W. A.; Murzin, V.; Kalinko, A.; Görlitz, M. High-flux XAFS-beamline P64 at PETRA III. *AIP Conf. Proc.* **2019**, *2054*, 060031, DOI: 10.1063/1.5084662.

(9) Ravel, B.; Newville, M. ATHENA, ARTEMIS, HEPHAESTUS: data analysis for X-ray absorption spectroscopy using IFEFFIT. *J. Synchrotron Radiat.* **2005**, *12*, 537–541, DOI: 10.1107/S0909049505012719.

(10) Battocchio, C.; Fratoddi, I.; Fontana, L.; Bodo, E.; Porcaro, F.; Meneghini, C.; Pis, I.; Nappini, S.; Mobilio, S.; Russo, M. V.; Polzonetti, G. Silver nanoparticles linked by a Pt-containing organometallic dithiol bridge: study of local structure and interface by XAFS and SR-XPS. *Phys. Chem. Chem. Phys.* **2014**, *16*, 11719–11728, DOI: 10.1039/C4CP01264J.

(11) Kesavan, J. K.; Luisetto, I.; Tuti, S.; Meneghini, C.; Battocchio, C.; Iucci, G. Ni supported on YSZ: XAS and XPS characterization and catalytic activity for CO2 methanation. *J. Mater. Sci.* **2017**, *52*, 10331–10340, DOI: 10.1007/s10853-017-1179-2.

(12) Panfil, Y. E.; Shamalia, D.; Cui, J.; Koley, S.; Banin, U. Electronic coupling in colloidal quantum dot molecules; the case of CdSe/CdS core/shell homodimers. *J. Chem. Phys.* **2019**, *151*, 224501, DOI: 10.1063/1.5128086.

(13) Adachi, S. *Properties of semiconductor alloys: Group-IV, III-V and II-VI semiconductors*; Wiley series in materials for electronic & optoelectronic applications; Wiley: Chichester U.K., 2009; DOI: 10.1002/9780470744383.

(14) Xie, R.; Kolb, U.; Li, J.; Basché, T.; Mews, A. Synthesis and characterization of highly luminescent CdSe-core CdS/$Zn_{0.5}Cd_{0.5}S$/ZnS multishell nanocrystals. *J. Am. Chem. Soc.* **2005**, *127*, 7480–7488, DOI: 10.1021/ja042939g.

(15) Adachi, S. *Properties of group-IV, III-V and II-VI semiconductors*; Wiley series in materials for electronic and optoelectronic applications; Wiley: Chichester, 2005; DOI: 10.1002/0470090340.

(16) Wei, S.-H.; Zunger, A. Calculated natural band offsets of all II–VI and III–V semiconductors: Chemical trends and the role of cation d orbitals. *Appl. Phys. Lett.* **1998**, *72*, 2011–2013, DOI: 10.1063/1.121249.

(17) Kalt, H.; Klingshirn, C. F. *Semiconductor Optics 1*; Springer International Publishing: Cham, 2019; DOI: 10.1007/978-3-030-24152-0.

(18) Madelung, O. *Semiconductors: Data Handbook*; Springer Berlin Heidelberg: Berlin, Heidelberg, 2004; DOI: 10.1007/978-3-642-18865-7.

(19) Hinuma, Y.; Grüneis, A.; Kresse, G.; Oba, F. Band alignment of semiconductors from

density-functional theory and many-body perturbation theory. *Phys. Rev. B* **2014**, *90*, 155405, DOI: 10.1103/PhysRevB.90.155405.
